# Lossless Compressed Sensing of Photon Counts for Fast Diffuse Correlation Spectroscopy

**DOI:** 10.1109/access.2022.3228439

**Published:** 2022-12-12

**Authors:** ARINDAM BISWAS, ASHWIN B. PARTHASARATHY

**Affiliations:** Department of Electrical Engineering, University of South Florida, Tampa, FL 33620, USA

**Keywords:** Biomedical computing, biophotonics, data compression, diffuse optics, diffuse correlation spectroscopy, optoelectronic sensors, photon counting

## Abstract

Diffuse Correlation Spectroscopy (DCS), a noninvasive optical technique, measures deep tissue blood flow using avalanche photon counting modules and data acquisition devices such as FPGAs or correlator boards. Conventional DCS instruments use in-processor counter modules that consume 32 bits/channel which is inefficient for low-photon budget situations prevalent in diffuse optics. Scaling these photon counters for large-scale imaging applications is difficult due to bandwidth and processing time considerations. Here, we introduce a new, lossless compressed sensing approach for fast and efficient detection of photon counts. The compressed DCS method uses an array of binary-coded-decimal counters to record photon counts from 8 channels simultaneously as a single 32-bit number. We validate the compressed DCS approach by comparisons with conventional DCS in experiments on tissue simulating phantoms and in-vivo arm cuff occlusion. Lossless compressed DCS was implemented with 87.5% compression efficiency. In tissue simulating phantoms, it was able to accurately estimate a tissue blood flow index, with no statistically significant difference compared to conventional DCS. Compressed DCS also recorded blood flow in vivo, in human forearm, with signal-to-noise ratio and dynamic range comparable to conventional DCS. Lossless 87.5% efficient compressed sensing counting of photon counts meets and exceeds benchmarks set by conventional DCS systems, offering a low-cost alternative for fast (~100 Hz) deep tissue blood flow measurement with optics.

## I. INTRODUCTION

Blood flow (BF) is a biomarker for tissue health because it is an indicator of metabolism and disease state in different parts of the body [1], [2], [3], [4], [5]. In recent years, Diffuse Correlation Spectroscopy (DCS) [6], [7], [8], [9] has emerged as a popular method for portable, noninvasive, bedside monitoring of deep tissue blood flow. DCS senses and quantifies an index of blood flow (*F*) in tissue microvasculature from intensity fluctuations in coherent laser light that has diffused through tissue. DCS blood flow indices have been validated against a variety of gold standard modalities including Doppler ultrasound [10], computed tomography (CT) [11] and Magnetic Resonance Imaging (MRI) [12]. DCS’s utility has been demonstrated for noninvasive deep-tissue blood flow measurements in adult/pediatric brain [8], [10], [13], [14], [15], in muscle [16], [17] and in spinal cord [18], [19], [20].

**FIGURE 1.**
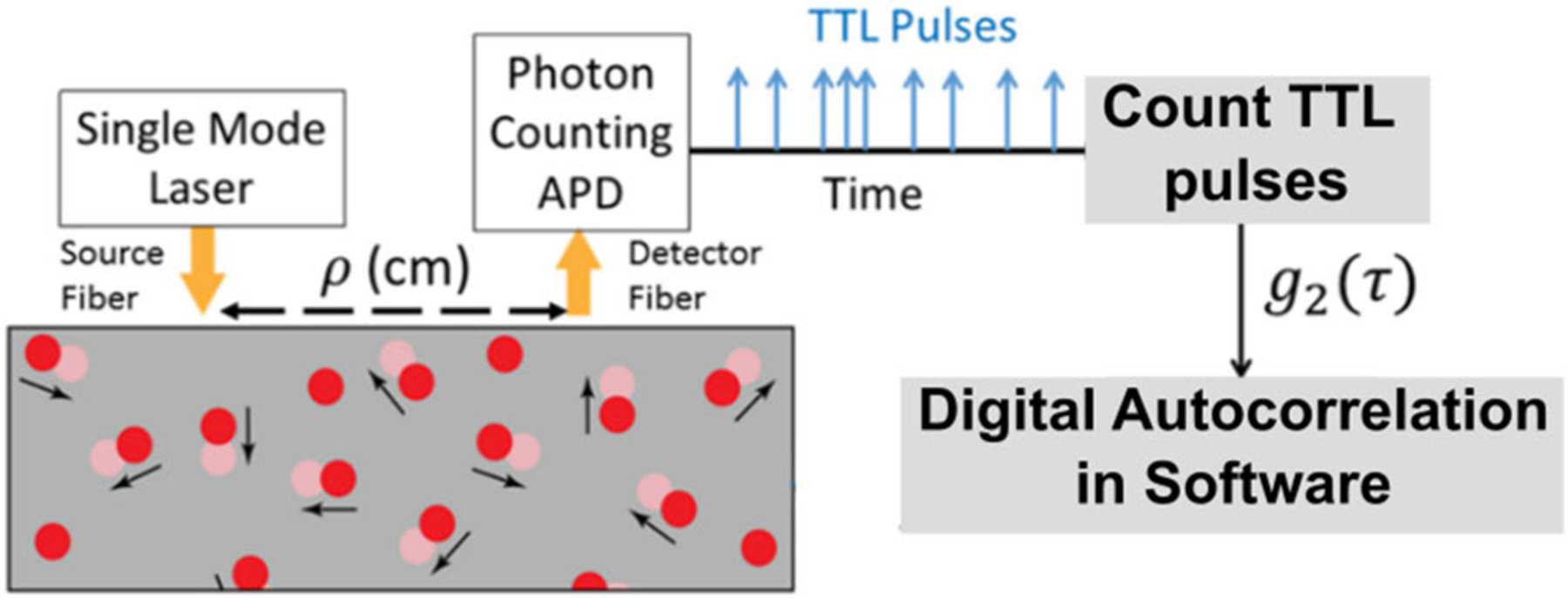
Schematic outlining Diffuse Correlation Spectroscopy. A wavelength stabilized laser is configured as an illumination source. Dynamic scatterers in the tissue (i.e., red blood cells) cause deviation in individual photon travel path resulting in temporal intensity fluctuations at the detector. These fluctuations are sensed by a series of APDs and converted to TTL pulses. A counter module is configured to sample these TTL pulses. The autocorrelation function g_2_(*τ*) of this sampled vector is fit to a correlation diffusion model to calculate the blood flow.

A typical DCS instrument, outlined in Fig. 1, comprises of a long coherence laser to illuminate the tissue, and single photon counting detectors to record light reflected from tissue. A custom correlator samples TTL pulses generated by the detectors (typically at 1–10 MHz) and computes an intensity autocorrelation function that quantifies temporal fluctuations in the light. Recently, we [21] and others [22], [23], [24] have demonstrated the use of customized National Instruments (NI) counter/timers or Field Programmable Gate Arrays (FPGAs) for acquisition, and software computation of DCS blood flow indices at speeds up to 100 Hz. These improvements to the temporal resolution have facilitated important new measurements such as noninvasive quantification of cerebral autoregulation [25], [26], [27], critical closing/intracranial pressure [28], [29], [30], and arteriole compliance [28], [31] in the brain. In DCS, single mode fibers are used to sample light from one speckle, which prevents speckle averaging at the detector and expands the dynamic range of measured intensity autocorrelation functions. However, since single-mode detection comes at the expense of signal levels, DCS intensity autocorrelation functions measured at a single channel are noisy. To overcome this limitation, typical implementations of DCS instruments often have more than one detection channel (3–6) at a single measurement site (or for a single source-detector separation) to improve signal-to-noise ratio of measured autocorrelation functions and hence blood flow estimates. Expansion of DCS measurements to an imaging configuration (e.g., Diffuse Correlation Tomography [32], [33]) would multiply the need for detection channels. For example, tomography with eight detection positions would require 32–48 detection channels. There are two major technical limitations that restrict use of multiple detection channels in DCS. Most commercial data acquisition systems limit the number of available counters/timers on a single board to 16. More significantly, parallel counting of photons and parallel computation of intensity autocorrelation functions from multiple detection channels will be a memory and computationally expensive process. These requirements for multiple digital counting elements (i.e., counters), increase instrument cost, complexity, and data bandwidth.

In this contribution, we introduce a new approach – compressed Diffuse Correlation Spectroscopy (compressed DCS) – for fast, computationally efficient, multi-channel measurement of DCS intensity autocorrelation functions without the use of processor embedded counter modules. Our approach implements photon counting using eight 4-bit Binary Coded Decimal (BCD) counters rather than digital in- processor (32-bit) counter modules typically used in DCS data acquisition systems. The compressed DCS system achieves an 87.5% data compression without compromising on measurement accuracy or signal-to-noise ratio, while maintaining low data burden and cost. In the following sections, we briefly describe the traditional DCS technique and the compressed DCS approach. We present experimental validation of the compressed DCS approach comparing it with a conventional eight-channel DCS system in both tissue simulating phantoms and in-vivo experiments on humans.

## II. DCS: THEORY AND BACKGROUND

A schematic of a typical DCS system is shown in Fig. 1. Light from a long coherence length near infrared laser source illuminates the tissue through an optical fiber. NIR light diffuses through tissue and is detected by a single mode optical fiber positioned 1–3 cm away from the source and is redirected to Single Photon Counting Avalanche Photodiode modules (APDs), that produce a TTL pulse for each detected photon [21]. Due to high temporal coherence of the laser source, changes in the optical pathlength of light diffusing through the tissue (i.e., due to scattering off moving particles/red blood cells) impart fluctuations in the intensity recorded at the detector, that are then used to compute the digital normalized intensity autocorrelation function. Blood flow is estimated by fitting the computed autocorrelation function to a diffusion model appropriate for the tissue geometry [7]. Photon counts are detected as a stream of TTL pulses, which are sampled by counter/timer modules at a fixed sampling frequency *f*_*s*_. If we consider the stream of photon counts as *n*(*i*), then the normalized intensity autocorrelation function, *g*_2_ (*τ*), is:

(1) $$g_{2}\left(\Delta n=\tau f_{s}\right)=\frac{\langle n(i)n(i+\Delta n)\rangle }{\langle n(i)n(i)\rangle }$$

Here, *τ* is the autocorrelation delay time and Δ*n* = *τf*_*s*_ is the integer number of shifts of photon count vector for a given delay time. The angle brackets (〈〉) indicates averaging of the autocorrelation function over a duration denoted by the integration time *t*_*int*_, which determines the overall speed of the measurement. For example, a system with 10 *ms* integration time will yield a 100 *Hz* acquisition rate. The size of individual photon count vector is defined by the integration time *t*_*int*_ and sampling time *t*_*sample*_. For, *t*_*int*_ = 10*ms* and *t*_*sample*_ = 1/*f*_*s*_ = 1*μs*, *n*(*i*) is a 10, 000 point vector. The measured intensity autocorrelation function (*g*_2_(*τ*)) is related to the normalized electric field autocorrelation function (*g*_1_ (*τ*)) through Siegert relation: [34]

(2) $$g_{2}(\tau )=1+\beta {\left|g_{1}(\tau )\right|}^{2}$$

where, *β* is an instrumentation factor that depends on light polarization, detector size and speckle size. For homogeneous semi-infinite tissue geometry, Correlation Diffusion theory [6] provides an analytical expression for *g*_1_ (*τ*) = *G*_1_(*τ*)/*G*_1_(0), where *G*_1_(*τ*) is the electric field autocorrelation function given by:

(3) $$G_{1}(\rho ,\tau )=\frac{3}{4\pi l_{tr}}\left[\frac{\text{exp}\left(-K(\tau )r_{1}\right)}{r1}-\frac{\text{exp}\left(-K(\tau )r_{b}\right)}{r_{b}}\right]$$

Here, $l_{tr}=1/\left(\mu_{a}+\mu_{s}^{\prime }\right)$ is the transport mean free path, $r_{1}={\left(l_{tr}^{2}+\rho^{2}\right)}^{0.5}$, $r_{\text{b}}={\left({\left(2z_{b}+l_{tr}\right)}^{2}+\rho^{2}\right)}^{0.5}$, *ρ* is the source-detector separation, *μ*_*a*_ is the tissue absorption coefficient, $\mu_{s}^{\prime }$ is the tissue reduced scattering coefficient, *z*_*b*_ = 2*l*_*tr*_(1+*R*_*eff*_)/3(1−*R*_*eff*_), and *R*_*eff*_ is the Fresnel effective reflection coefficient. *K*(*τ*) is a dynamic wave-vector that depends on the blood flow index (*F*); $K(\tau )={\left[3\mu_{a}\left(\mu_{a}+\mu_{s}^{\prime }\right)\left(1+2\mu_{s}^{\prime }k_{0}^{2}F\tau /\mu_{a}\right)\right]}^{0.5}$ and *k*_0_ = 2*π*/λ. The acquired intensity autocorrelation function is fit to this model to compute *F*. In a typical implementation, DCS photon counts are sampled and recorded using in-processor Counter/Timer modules (for example, using a National Instruments PCIe/PXIe6612 board with eight counter/timers in [21]). DCS intensity autocorrelation functions were computed in software (LabVIEW) and blood flow indices were estimated offline.

## III. COMPRESSED DCS: MULTIPLE CHANNEL SAMPLING WITH BCD ARRAY

Here, we outline our approach for compressed DCS, using custom electronics for memory efficient recording of photon counts. Prior implementations of DCS involved use of in-processor counter/timer modules (typically 32-bit). For example, we and others have used 32-bit counters on an NI PCIe/PXIe-6612 or similar boards. Recall, that DCS detects light using single mode fibers with output powers in the order of a few pW; the typical photon count rate in a DCS measurement ranges from 10–1000 kHz in one channel, or even up to 2–4 MHz on the higher end. Considering the higher end of the photon count rates, at a sampling frequency of 1 MHz (*t*_*sample*_ = 1*μs*), these intensity levels result in counts of either 0, 1 or 2 for each microsecond, i.e., elements of the vector *n*(*i*) are either 0, 1 or 2. Using a 32-bit counter for these low-light (low-count) applications is highly inefficient. Indeed, only 1/16 or 1/32 of the data capacity of the 32-bit register is used when counts are 0, 1 or 2 and only 2 least-significant bits of the 32-bit counter change with each sampling. In other words, a maximum photon count rate of 2 MHz can be represented with just two data bits of an incremental counter capable of storing minimum of 2-bits. With 32-bit counters most of the memory and data communication bandwidth is largely underutilized. We further note that for systems/experiments with faster sampling rate (e.g., *t*_*sample*_ = 100 *ns*), these inefficiencies worsen.

**FIGURE 2.**
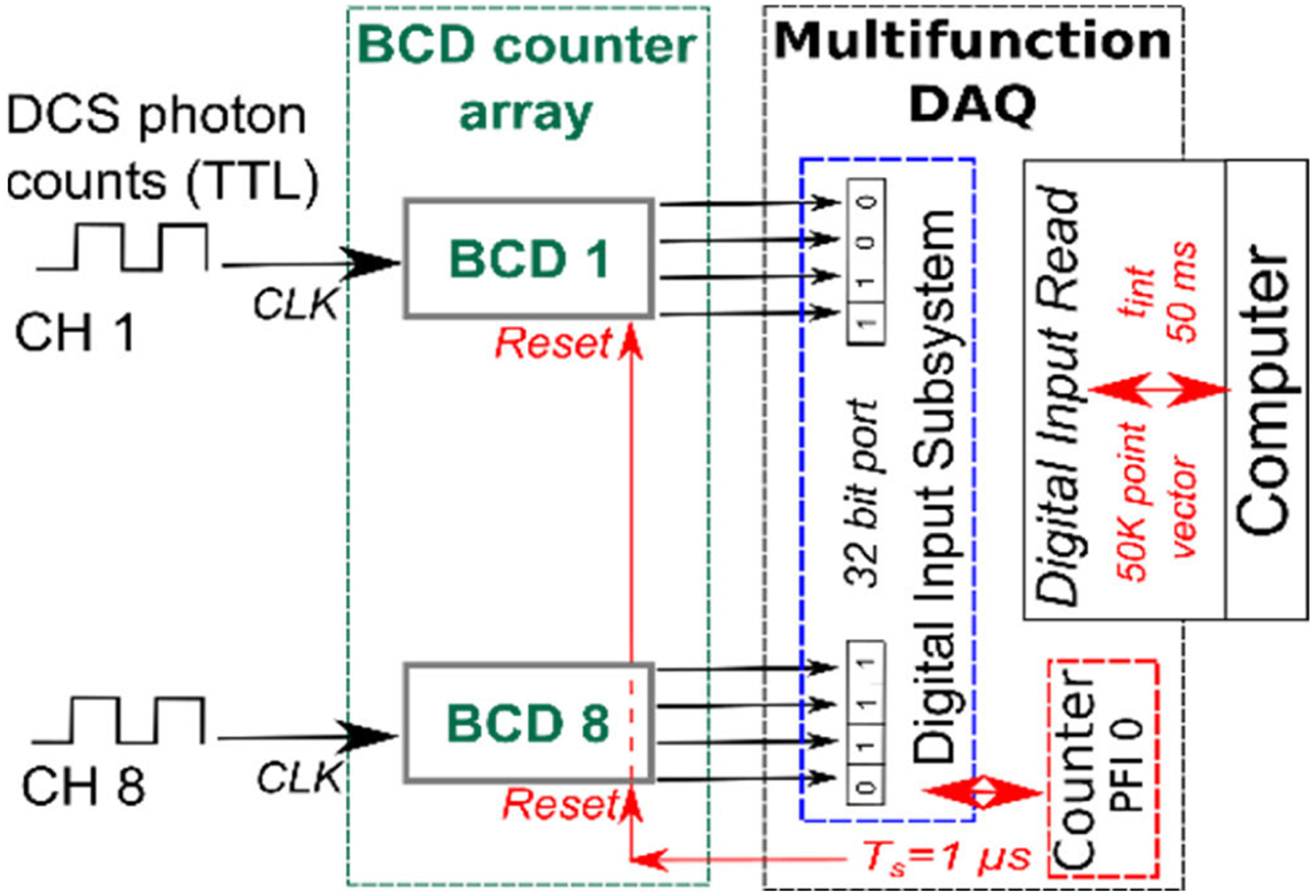
Block diagram outlining the compressed DCS photon counting system. The outputs from individual single photon counting APDs were routed to the clock pin (CLK) of a BCD counter. The output from the 8-BCD counters (i.e., 4-bit output for each BCD counter for a total 32-digital lines) was sampled through a 32-bit digital I/O port of a multifunction DAQ and recorded on a computer for processing. A timer from DAQ (i.e., NI PCIe-6353) was configured to reset all BCD counters at a frequency of *f*_*s*_ = 1*MHz*. For an integration time of *t*_*int*_ = 50*ms* a total of 50, 000 sample 32-bit vector was recorded.

Practically, this compressed DCS is implemented using widely available low-cost BCD chips (e.g., 74LS90). The schematic of one such implementation is shown in Fig. 2. BCD counters use 4-bits to store decimal values up to 10. The TTL outputs from a single photon counting module are connected to the clock input of the BCD counter, which increments its internal register with every TTL pulse (photon). Thus, an array of eight BCD counters can simultaneously sample DCS photon counts from eight single photon couniting modules which together form a single 32-bit integer output (i.e., 4-bit output of 8 BCD counter). The 32-bit integer is recorded via general purpose digital input/output lines of a multifunction data acquisition system (National Instruments PCIe-6353). The data acquisition process is controlled by two software-controlled counter/timers on the data acquisition board. The first, operating at 1MHz clocks the digital I/O read operation, and simultaneously resets the BCD counters. The second controls the integration/averaging time of the photon counts. Thus, an integration time of *t*_*int*_ = 50 *ms* would yield a 50K point vector of photon counts. Custom software (LabVIEW) is used to control the data acquisition process and perform bit-wise operations to separate photon counts from individual channels (i.e., combination of 4-bits) to compute the DCS intensity autocorrelation functions as described earlier [21]. For a single channel, the compressed DCS system utilizes 4-bits to record photon counts, compared to the conventional DCS system which uses 32 bits. This gives our compressed DCS system a data compression efficiency of 87.5%.

## IV. EXPERIMENTS AND RESULTS

All experiments were carried out with a custom DCS instrument. Briefly, light from a wavelength stabilized laser (Toptica Photonics, iBeam Smart, 785nm, 120mW, coherence length >50m) was coupled to a multi-mode fiber and used to illuminate the sample (i.e., tissue phantom or human forearm). Reflected light from the sample was collected using single mode fibers placed 1 cm and 2.5 cm away from the source and redirected to single photon counting APD modules (Excelitas, SPCM-AQ4C); three detection channels were used at 1 cm source-detector separation, while five detector channels were used for the 2.5 cm source detector separation. All fibers were set in place using a custom silicon mold to create an optical probe as described earlier [21], [35]. TTL outputs from each single photon counting APD were directed to both the BCD counter array (for compressed DCS software autocorrelation measurements as described in Section III) and 32-bit counters on an NI-9174/NI-9401 for conventional DCS autocorrelation measurement with a software correlator [21], [35]. In both cases, autocorrelation functions recorded from the same source-detector separation were averaged.

### A. INTENSITY AUTOCORRELATION FUNCTIONS MEASURED WITH COMPRESSED DCS: VALIDATION ON A SOLID TISSUE SIMULATING PHANTOM

**FIGURE 3.**
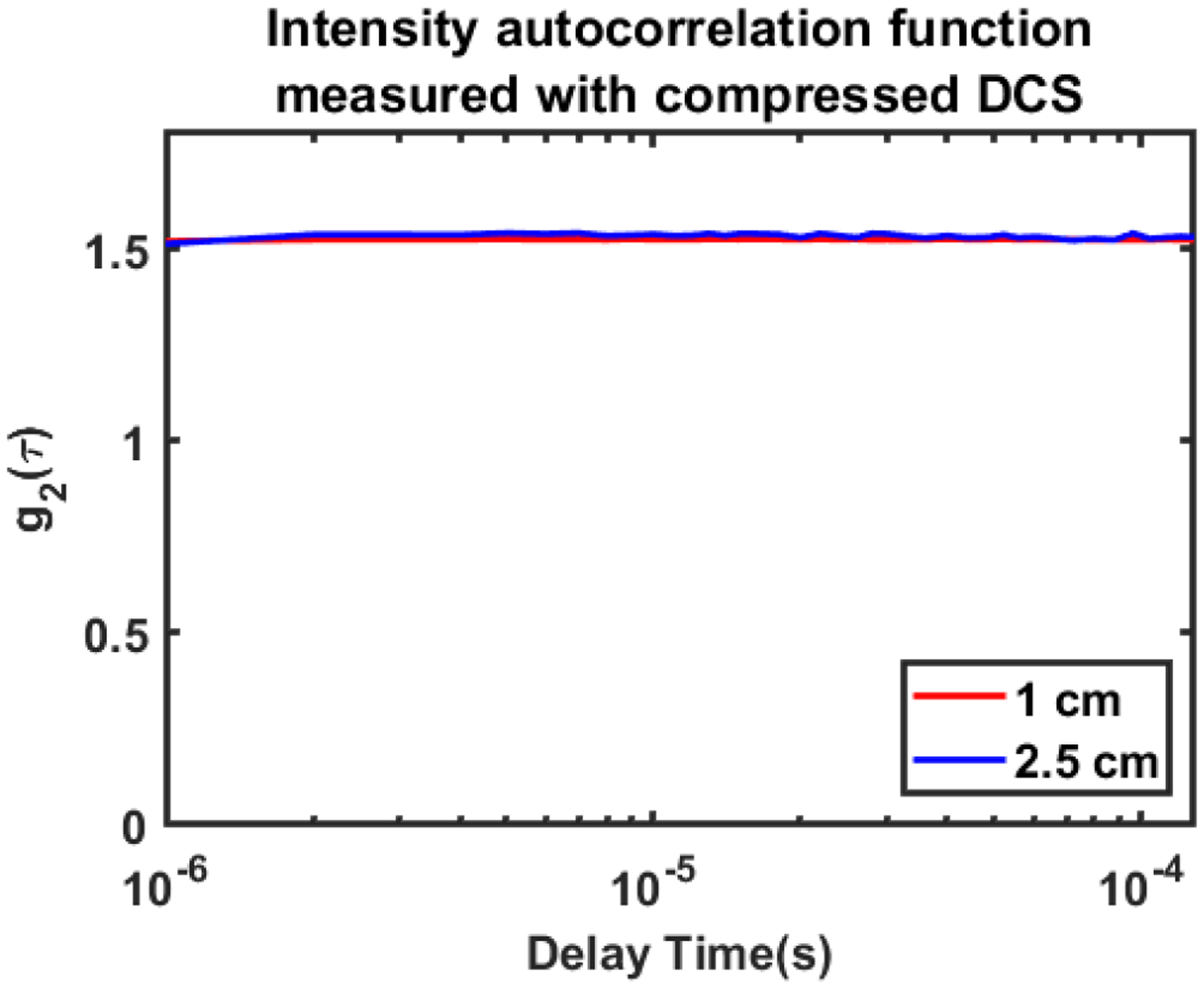
DCS intensity autocorrelation curves g_2_(*τ*) acquired from a solid phantom using the compressed DCS system at source-detector separations of 1 cm (red curve) and 2.5 cm (blue curve). The solid phantom had optical properties of *μ*_*a*_ = 0.07 *cm*^−1^ and $\mu_{s}^{\prime }=10\;cm^{-1}$ at 850 nm. A total of 100 g_2_(*τ*) curves were averaged for these plots.

We first demonstrate the ability of the compressed DCS system to acquire and compute autocorrelation curves from a solid tissue simulating phantom. For this experiment the DCS probe was secured to the surface of solid phantom (110*mm* × 110*mm* × 45*mm*) with absorption coefficient *μ*_*a*_ = 0.07 *cm*^−1^ and reduced scattering coefficient $\mu_{s}^{\prime }=10.7\;cm^{-1}$ at 850 nm (INO Biomimic Phantoms, Quebec, CA). The phantom was illuminated with a surface optical power of 72 mW. The compressed DCS software correlator was configured to a sampling frequency of *f*_*sampling*_ = 1 *MHz*, and an integration time of *t*_*int*_ = 100 *ms*, resulting in an effective acquisition frequency of 10 *Hz*. Each frame (i.e., photon counts recorded over 100 *ms* integration time) contained 100,000-point vector of 32-bit data for a single detection channel. For each channel autocorrelation function was calculated with digital shifts of 1 to 250 samples, corresponding to delay times (*τ*) of 1*μs* to 250 *μs*. Fig.3 shows an average of 100 DCS intensity autocorrelation functions measured from a solid phantom at 1 and 2.5 cm source detector separation (red and blue curves respectively) using the compressed DCS software correlator. The autocorrelation curves do not decay, indicating no dynamic fluctuations (as expected with a solid phantom). These results offer the first validation of the compressed DCS system.

### B. INTENSITY AUTOCORRELATION FUNCTIONS AND FLOW INDICES MEASURED WITH COMPRESSED DCS: VALIDATION ON A LIQUID SIMULATING PHANTOM

**FIGURE 4.**
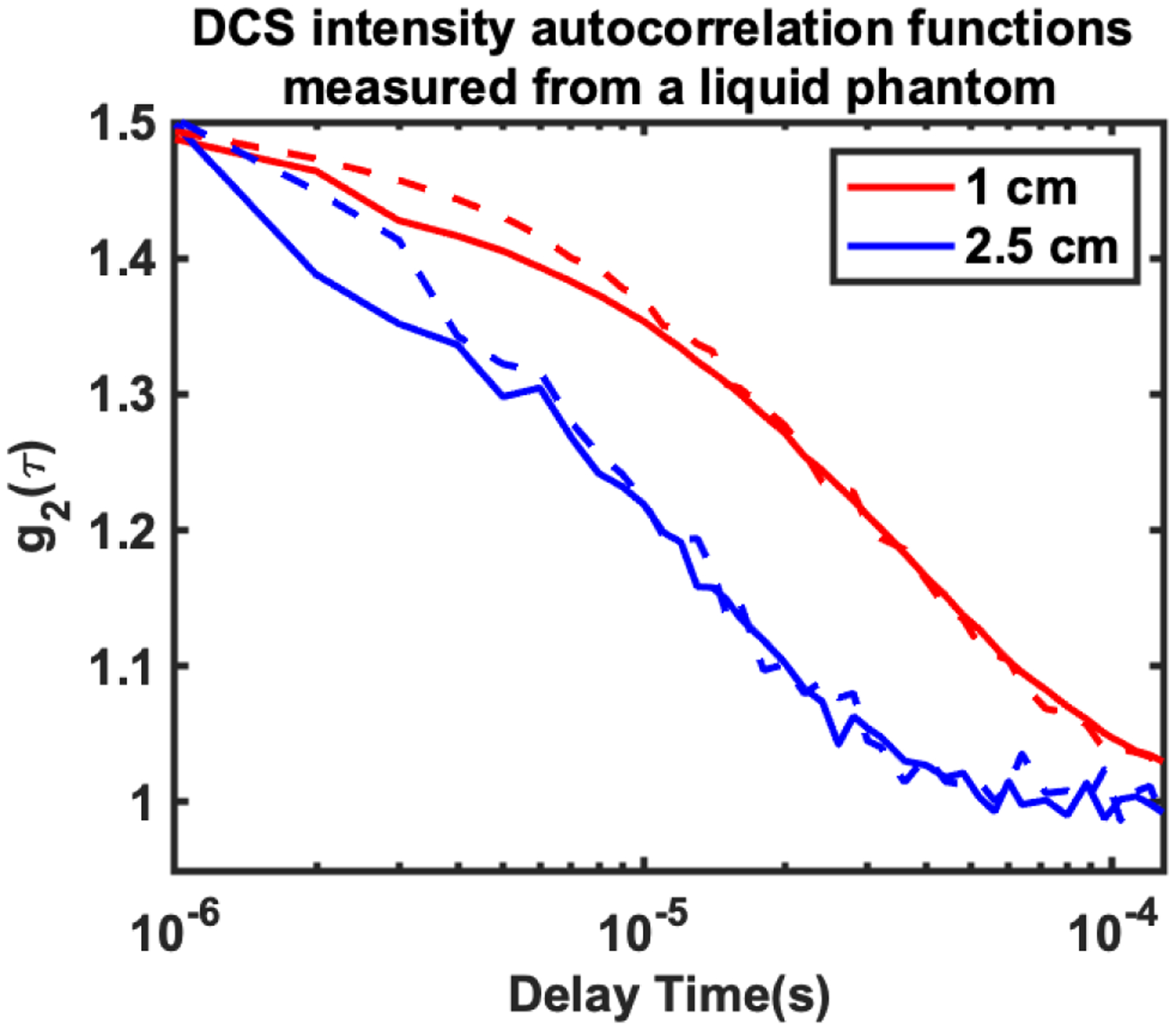
DCS intensity autocorrelation function (g_2_(*τ*)) acquired from a tissue simulating liquid phantom using the compressed DCS (solid lines) and conventional DCS (dashed lines) systems at 1 cm (red) and 2.5 cm (blue) source detector separations. The liquid phantom had optical properties of *μ*_*a*_ = 0.1 *cm*^−1^ and $\mu_{s}^{\prime }=10\;cm^{-1}$ at 785 nm. Results shown are an average of 70 curves each recorded at an integration time of 100 ms.

We next demonstrate the accuracy of flow estimates measured with the compressed DCS system, by comparing it to conventional DCS autocorrelation measurements on a liquid tissue simulating phantom. A tissue simulating liquid phantom was prepared from Intralipid (20% emulsion, Sigma-Aldrich, MO), India ink and distilled water, to realize a sample with absorption coefficient *μ*_*a*_ = 0.1 *cm*^−1^ and reduced scattering coefficient $\mu_{s}^{\prime }=10\;cm^{-1}$ at 785 nm. The DCS probe was placed on the surface of the liquid phantom, and DCS intensity autocorrelation functions were recorded using both traditional DCS and compressed DCS at 10 Hz acquisition rate. Fig. 4 shows representative intensity autocorrelation functions acquired by the compressed DCS system (solid lines) from the liquid phantom at 1 cm (red line) and 2.5 cm (blue line) source detector separations. These curves represent an average of 70 *g*_2_(*τ*) curves, each acquired with an integration time of 100 ms. Here, the decay in the autocorrelation function is caused by fluctuations in the photon intensity that manifest dynamic scattering from Brownian motion of fat molecules in the intralipid. The decay at 2.5 cm source detector separation is faster than at 1 cm because increased photon travel length at the higher source-detector separation allows for more dynamic scattering to influence the intensity fluctuations. The autocorrelation functions measured with compressed DCS compare favorably to those measured with conventional DCS system (dashed lines), with similar decay rates.

**FIGURE 5.**
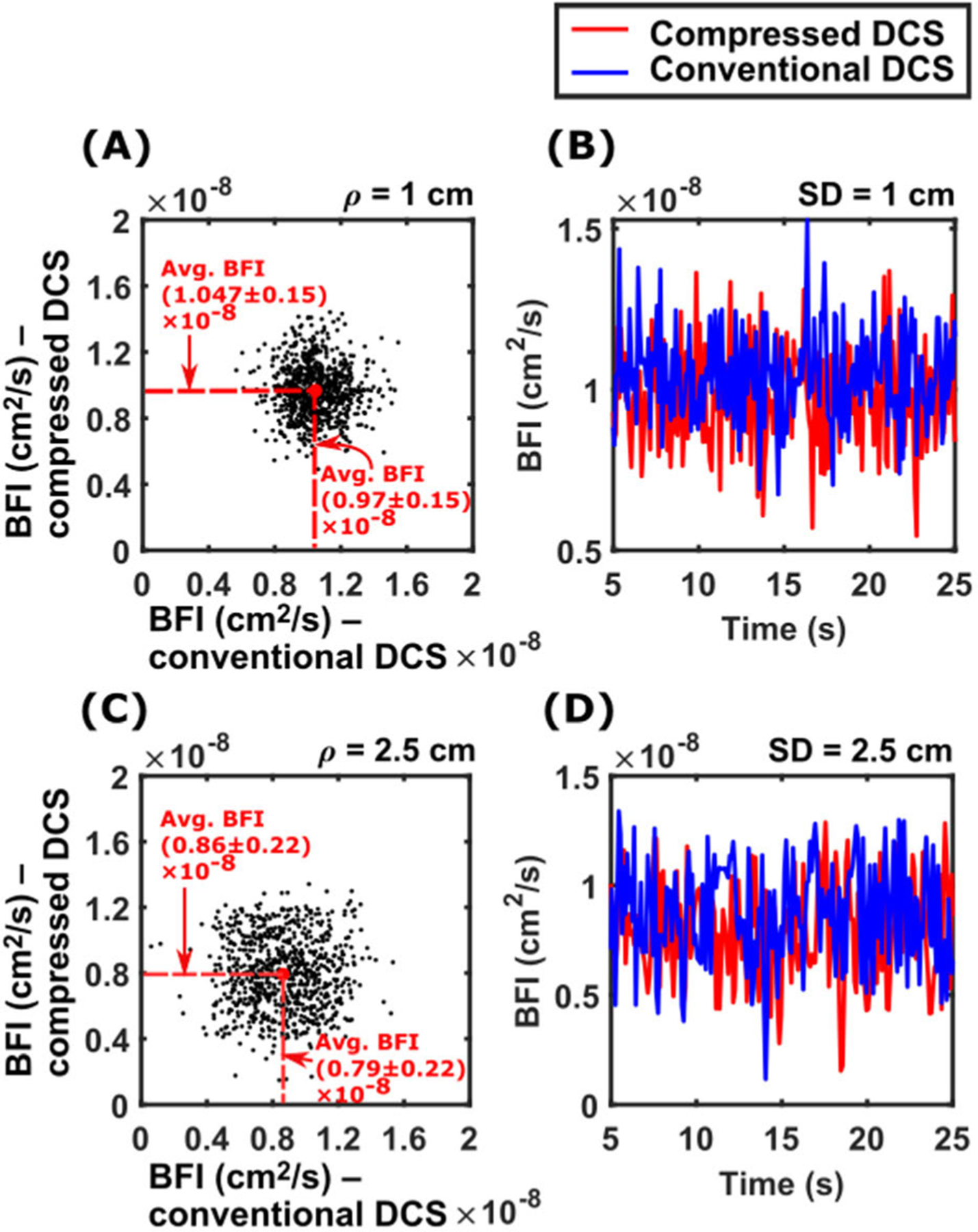
Comparison of blood flow indices estimated with compressed and conventional DCS systems from a liquid phantom. (A) and (C) show the scatter plot of the flow indices estimated by compressed DCS (y-axis) and conventional DCS (x-axis) for source detector separations *ρ* = 1 *cm* and *ρ* = 2.5 *cm* respectively. (B) and (C) show the comparative temporal traces of flow index measured with compressed DCS (red curve) and conventional DCS (blue curve). Results show good 1:1 correspondence, including similar average flow indices (no statistically significant difference, two-sample t-test).

To further validate the compressed DCS system, we compared the blood flow indices estimated from the measured intensity autocorrelation function with those estimated using a conventional DCS instrument. Using the methods and probes described earlier, intensity autocorrelation functions were recorded simultaneously from the liquid phantom using both compressed DCS and conventional DCS instruments. DCS intensity autocorrelation functions measured at 10 Hz from source detector separations 1 cm and 2.5 cm were fit to the semi-infinite solution to the correlation diffusion equation (Eq. 3) to estimate *F*. Fig. 5 shows the results of this comparison. Fig. 5(A) and 5(C) show scatter plots of flow indices simultaneously measured using the two systems, for source-detector separations of 1 and 2.5 cm respectively. Here, flow index estimated with the compressed DCS system is in the y-axis, while those estimated with the conventional DCS system is in the x-axis. Fig. 5 (B) and (D) show the respective time courses of these flow indices, with the red curve depicting flow indices measured with compressed DCS and the blue curve depicting flow indices measured with conventional DCS. At 1 cm source-detector separation, the compressed DCS system estimated an average flow index of $F_{\text{cmpDCS}}^{1cm}=(1.047\pm 0.15)\times 10^{-8}cm^{2}/s$, while the conventional DCS system estimated an average flow index of $F_{\text{cnvDCS}}^{1cm}=(0.97\pm 0.15)\times 10^{-8}cm^{2}/s$. At 2.5 cm source-detector separation, the compressed DCS system estimated an average flow index of $F_{\text{cmpDCS}}^{2.5cm}=(0.86\pm 0.22)\times 10^{-8}cm^{2}/s$, while the conventional DCS system estimated an average flow index of $F_{\text{cnvDCS}}^{2.5cm}=(0.79\pm 0.22)\times 10^{-8}cm^{2}/s$. The errors represent the standard deviation of flow index estimates over the measurement period. A two-sample t-test revealed no statistically significant difference in the flow estimates estimated by the two instruments for both source-detector separations. These results validate the accuracy of the flow indices estimated by the compressed DCS system.

### C. SIGNAL-TO-NOISE RATIO OF AUTOCORRELATION FUNCTIONS MEASURED WITH COMPRESSED DCS: VALIDATION ON A LIQUID SIMULATING PHANTOM

**FIGURE 6.**
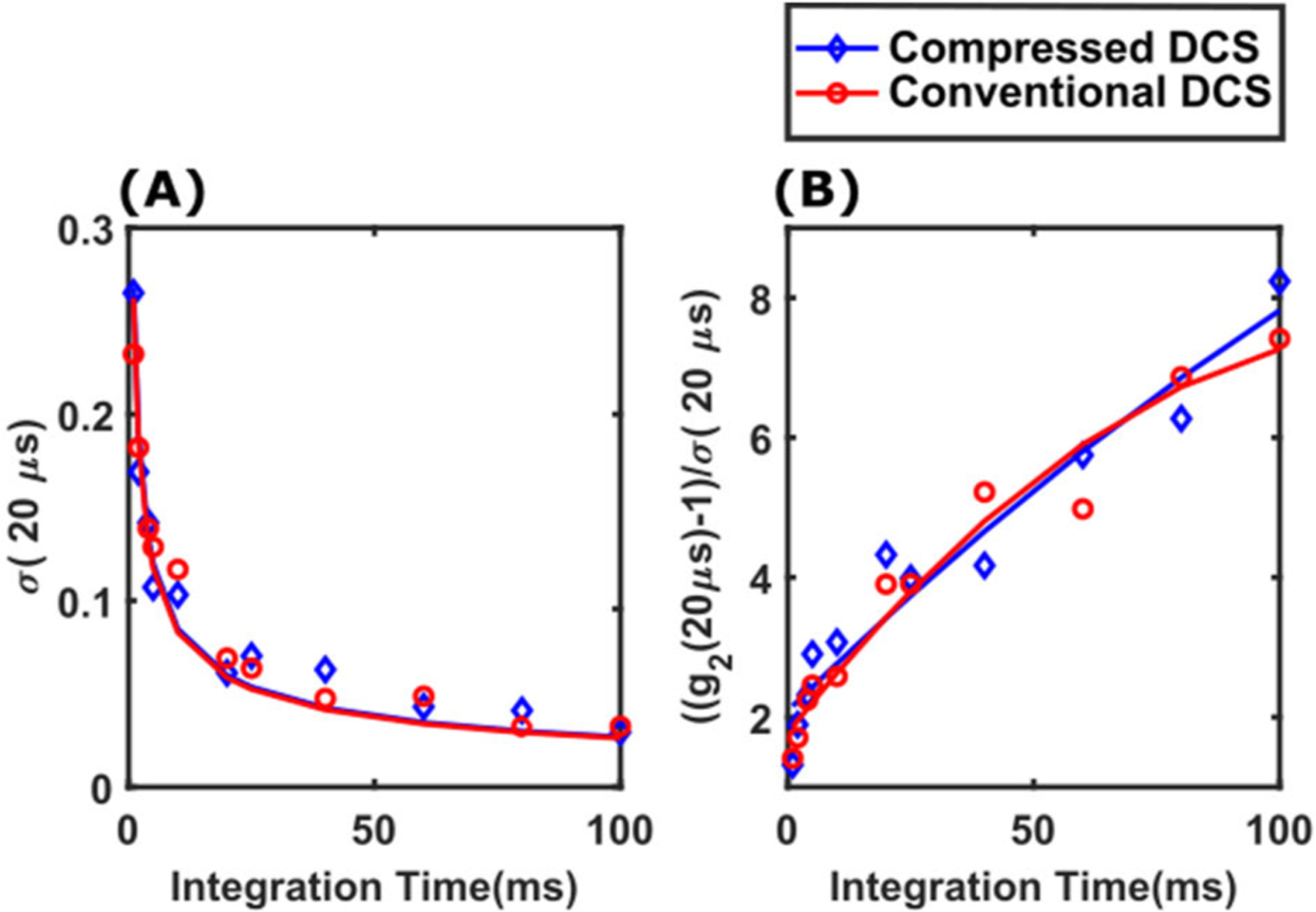
Noise (A) and Signal-to-Noise Ratio (B) of intensity autocorrelation functions measured using compressed DCS (blue) and conventional DCS (red) for representative delay time of 20*μs* Measurements were performed on a liquid phantom at source-detector separation of 1 cm and detection photon count rate of 100kHz. Markers show individual data points while the solid lines are fits to the DCS correlation noise model.

The final tissue phantom validation experiment concerns the comparison of the signal-to-noise ratios of intensity autocorrelation functions measured using compressed DCS and conventional DCS systems. Following the well-established DCS correlation noise model [32], we defined ‘noise’ (*σ*(*τ*)) as the standard deviation of the measured intensity autocorrelation function *g*_2_ (*τ*), and signal-to-noise ratio (SNR) as *ζ* (*τ*) = (*g*_2_ (*τ*) − 1)/*σ*(*τ*)). DCS intensity autocorrelation functions were recorded simultaneously from the liquid phantom (1 cm source-detector separation) using both compressed DCS and conventional DCS systems. Since SNR depends on signal intensity, the light intensity for the measurement was fixed such that the detectors recorded an average photon count rate of 100 kHz. Autocorrelation functions were acquired at different rates by varying the integration time (*t*_*int*_) of the measurements from 1 ms to 100 ms (i.e., acquisition frequency of 1000 Hz to 10 Hz). Fig. 6(A) shows the noise in measurement of autocorrelation function at 20*μs* delay time, i.e., *σ*(20 *μs*), measured with both compressed DCS (blue) and conventional DCS (red). Fig. 6(B) shows the corresponding SNR. In both cases, the markers represent measurements from the liquid phantom, while the solid lines represent fits of the noise/SNR measurement to the correlation noise model [32]. It is readily apparent that the noise model fits well with the measured noise/SNR data. Moreover, the noise/SNR of compressed DCS measurements are comparable to those recorded with conventional DCS. This is an important validation step, because it shows that the data compression effected by the BCD counters is not at the expense of measurement SNR. Here, 20*μs* has been selected as a delay time to illustrate the noise performance, because it represents to a section of the autocorrelation function that is sensitive to changes in flow (Fig. 4). These results are in-line with our previous experiments to characterize SNR of DCS systems [21], [35].

### D. DYNAMIC BLOOD FLOW MEASUREMENT IN HUMAN ARM WITH COMPRESSED DCS

**FIGURE 7.**
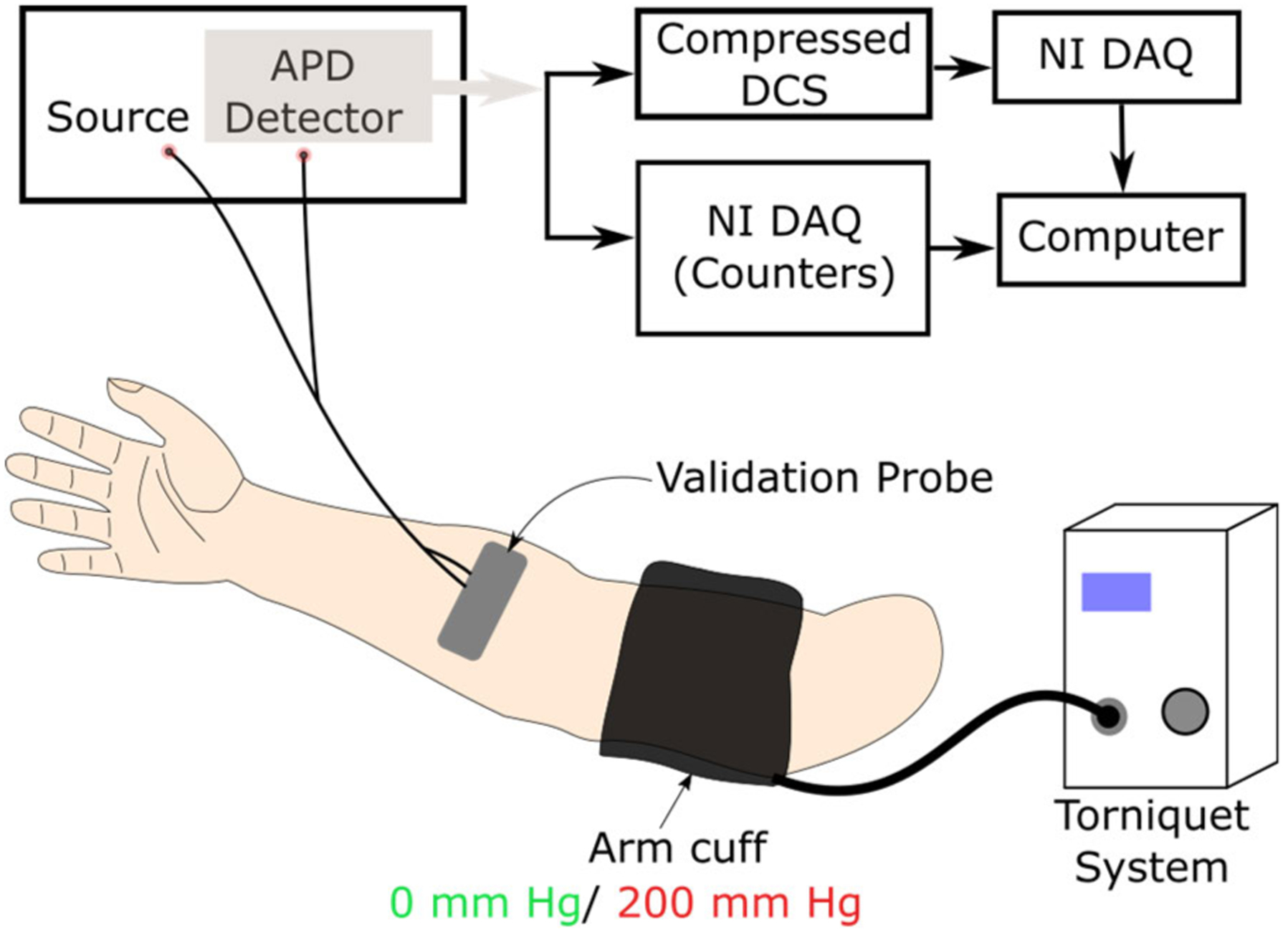
Schematic of in vivo arm-cuff occlusion experiment to validate compressed DCS blood flow measurements. The arm cuff was attached to the bicep muscle of the volunteer. The optical probe was placed on the forearm and was connected to the light source and detectors via optical fibers. The output of the detector (i.e., TTL pulse output) is connected in both compressed DCS system (i.e., BCD counter) and conventional DCS software correlator (i.e., NI DAQ counters). An automatic tourniquet system was used to inflate the arm cuff to 200 mmHg to effect occlusion.

Finally, we demonstrate and validate that the compressed DCS system can accurately measure blood flow changes *in vivo*. To this end, we measured the blood flow dynamics on a human forearm during arm cuff occlusion using both compressed DCS and conventional DCS instruments. *In vivo* experiments were approved by the Institutional Review Board of the University of South Florida (Protocol number Pro00039832_CR000002, approved 11/16/2021). Fig. 7 shows a schematic of the experiment. An optical probe (described earlier) was placed on the forearm of an adult volunteer (male, 25 years old), and was connected to the light source and single photon counting detectors using fiber optic cables. The output of the detector (i.e., TTL pulses for each detected photon) was connected to both the compressed DCS system (i.e., BCD counters) and a conventional DCS software correlator (NI-DAQ counters). Data acquisition was realized using custom LABVIEW software for both instruments. An arm cuff was wrapped over the subject’s bicep and was connected to an automatic pump (A.T.S. 4000, Zimmer, USA). The experiment protocol consisted of a 1-minute baseline, followed by a 1-minute occlusion period (arm-cuff inflated to 200 mmHg), and a 1-minute recovery period. Laser power was controlled to be within ANSI limits of safe exposure for skin [36]. DCS intensity autocorrelation functions were recorded at 10Hz using both systems, and a blood flow index was computed by methods described earlier.

**FIGURE 8.**
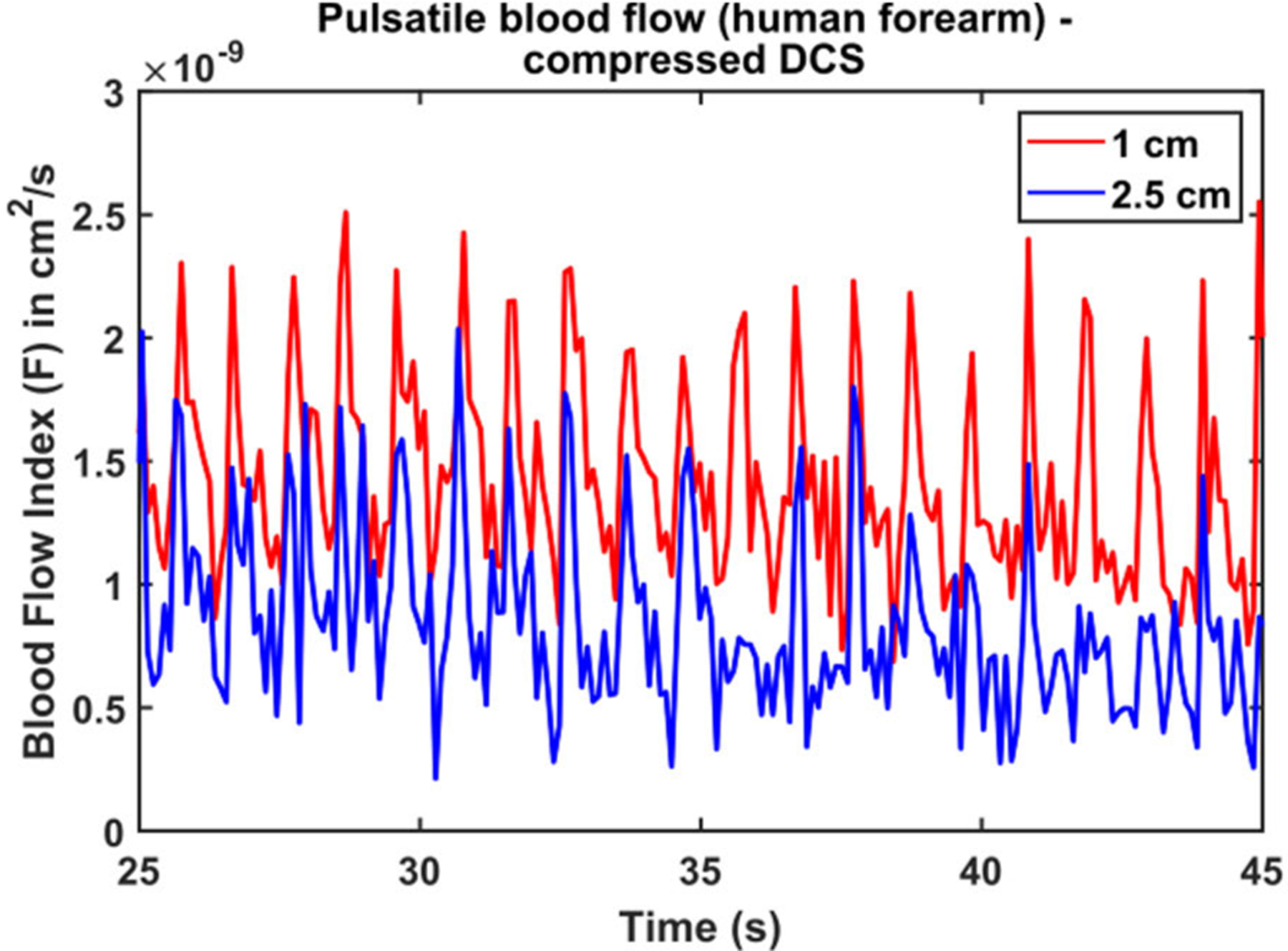
Pulsatile blood flow measured on a human forearm with compressed DCS. The red/blue lines indicate time courses of blood flow indices measured at source detector separations of 1 cm/2.5 cm. Both cases can clearly resolve flow changes similar to the QRS peak and the dicrotic notch.

Fig. 8 shows a representative time course of blood flow dynamics measured with the compressed DCS system under baseline conditions. Blood flow measured at both 1cm, and 2.5 cm source detector separation show dynamic blood flow changes; the pulsatility of blood flow due to the heartbeat is clearly resolved. In addition, some high frequency waveform features similar to the QRS peak and the dichrotic notch are well resolved; the dicrotic notch represents a brief increase in blood pressure and blood flow following closure of the aortic valve in the cardiac cycle. We note that that the noise in the measurements is greater at 2.5 cm source detector separation. This is to be expected as the measured photon count rates are lower for longer source-detector separation. The pulsatile blood flow measurements demonstrates that the compressed DCS system can resolve small flow changes and that it can be used for high-speed blood flow measurements. These results are similar to our prior work on pulsatile blood flow detection with conventional DCS systems [21], [35].

**FIGURE 9.**
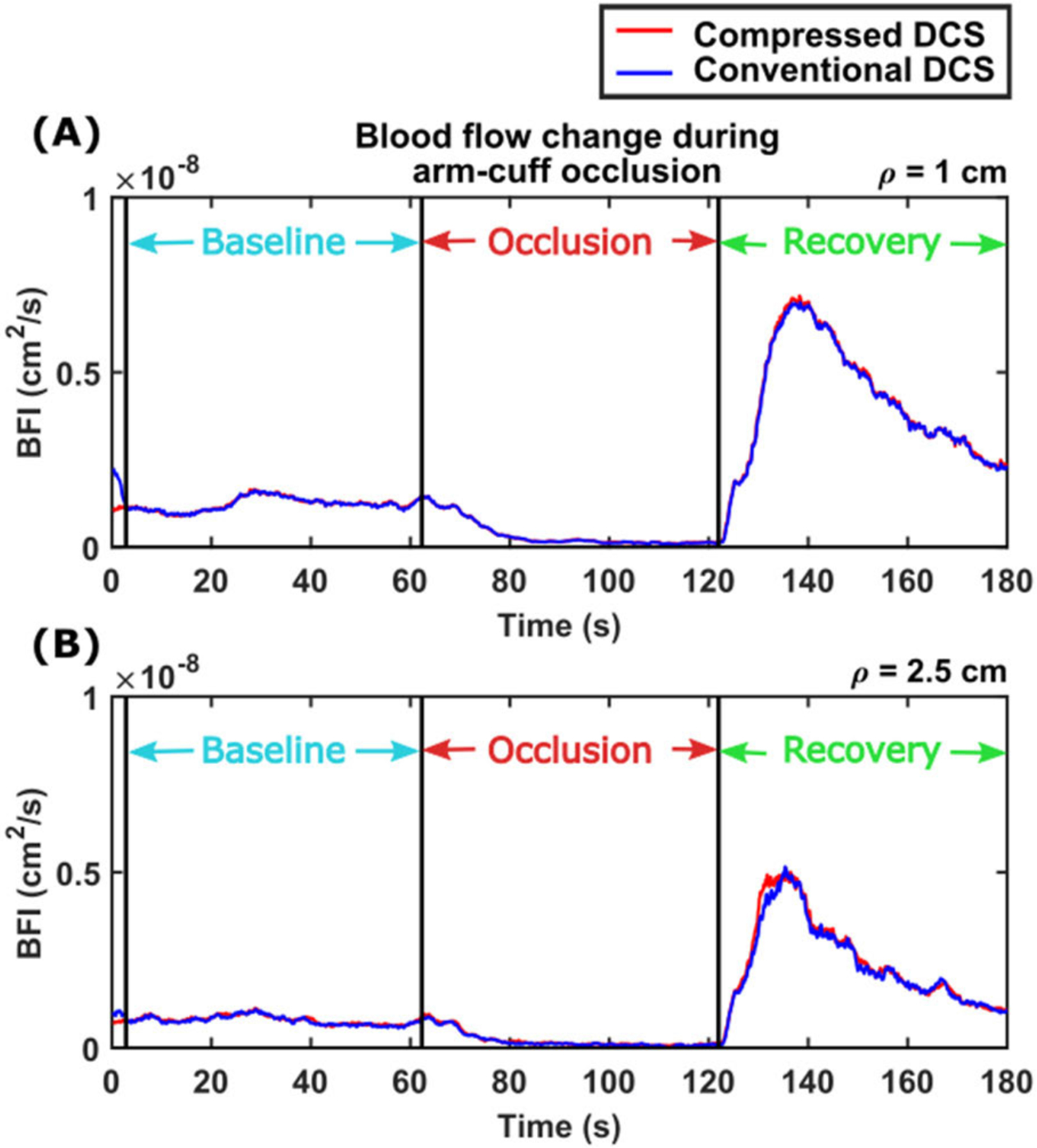
Quantitative changes in forearm blood flow measured during an arm-cuff occlusion with compressed (red curve) and conventional (blue curve) DCS systems. The time courses of blood flow indices are averaged with a 20-point (2 second) moving average window. (A) represents the blood flow changes measured at source-detector separation *ρ* = 1 *cm* and (B) represents the blood flow changes measured at source-detector separation *ρ* = 2.5 *cm*. Blood flow changes measured with compressed and conventional DCS systems are in good agreement with each other; both record almost 100% reduction in blood flow during occlusion, and a strong reperfusion response during the recovery period.

Fig. 9 shows the results of the arm-cuff occlusion experiment, for source-detector separations of 1 cm (Fig. 9A) and 2.5 cm (Fig. 9B). Time course of the blood flow index are displayed as a function of time during three phases of the experiment – baseline, occlusion, and recovery. Time courses of the blood flow index were smoothed with a 20-point (2-second) moving average window. The dynamics of blood flow changes are clearly visualized by both compressed DCS (red curve) and conventional DCS (blue curve). During the 1-minute occlusion phase, blood flow reduces by almost 100%, which is accurately measured with the compressed DCS system. Both compressed DCS and conventional DCS systems also track the reperfusion in blood flow in the recovery phase. Importantly, the data compression does not impact the estimated blood flow indices over a large flow change, showing that the compressed DCS has a dynamic range that is comparable to conventional DCS instruments.

## V. DISCUSSION

This article describes a new approach to high-speed sampling of DCS photon counts and fast software computation of intensity autocorrelation functions using resource- and cost-efficient data acquisition units. The key innovation is the development of a data compression approach (Section III) that identifies and exploits inefficiencies in conventional photon counting for DCS. The lossless data compression is 87.5% efficient and can be implemented using simple, low-cost digital circuits. We validated the accuracy of blood flow measured with the compressed DCS system with experiments on tissue simulating phantoms (Fig. 3–5). We further characterized and validated the signal-to-noise ratio of measured DCS intensity autocorrelation functions, by comparing the performance of the compressed DCS system to conventional DCS systems, and by fitting measurement noise to a DCS correlation noise model (Fig. 6). Finally, we performed *in vivo* validation of the compressed DCS system, by measuring the blood flow dynamics in a human forearm during arm-cuff occlusion. The demonstrations of blood flow pulsatility (Fig. 8) and measurements of larger blood flow changes (Fig. 9) *in vivo* highlight the sensitivity and dynamic range of the compressed DCS system. In all cases, performance of the compressed DCS system met benchmarks set by conventional DCS instruments.

The current implementation of the compressed DCS approach, used a generic multi-function data acquisition device to read photon-counts off the BCD counters as a stand-alone realization of the compressed DCS instrument. Critically, the data compression (photon counting with an array of BCD counters) can be readily implemented in other realization of fast DCS instruments, such as those that use FPGAs [22], [23], FFT-based software correlators [37], [38], or multi-core microcontroller units/MCUs (e.g., Texas Instrument TMS320F28379D, Infineon TC275Dx) that are capable of multithreaded operations for real-time sampling of DCS photon counts and computation of blood flow indices [39]. Since the data compression occurs at the photon counting stage, we expect that our approach will be just as effective in any implementation of DCS.

More generally, the data compression approach presented here can be extended to other high-speed photon counting applications, with optimizations to account for the highest expected photon count rate. Per optical techniques, typical photon counting APDs have a response time of 25 ns and a ‘dead-time’ of 50 ns, which translates to a maximum detectable count rate of 10–13 MHz (i.e., 10–13 counts/*μs*) [21] – this is within the capability of the BCD counter. Thus, the compressed photon counting approach can be readily adapted to other optical technologies such as Fluorescence Correlation Spectroscopy, time-domain near infrared spectroscopy, or fluorescence lifetime measurements.

We note a few design considerations and limitations of the compressed DCS approach. The current implementation of compressed DCS used 4-bits of the BCD counter to sample photon counts from one channel. Eight such channels form a single 32-bit number that is recorded via a digital I/O line. Scaling this approach to more channels would require the availability of several such digital lines. Practical implementations of these lines will be straightforward in FPGAs or dedicated digital I/O boards but will also require careful bit-wise operations to accurately parse the data, and sufficient data throughput to transfer the counts. The bandwidth required for transferring 32-bit integers (i.e., 8 channels) every *μs* over 1 second is ~3.8 MB/s. Note that the bandwidth requirements for conventional DCS systems in these cases would be 16 times greater. Furthermore, depending on the expected photon count rate, the bits per channel can be reduced to 2, which will help alleviate this limitation. Compared to a standard DAQ counter based acquisition system the compressed DCS system may experience some count loss (<2% at 1 *MHz* sampling frequency) due to the overlapping of TTL pulses with reset duration. However, our experimental results shows that this data loss doesn’t affect the calculation of autocorrelation functions or blood flow estimates. Finally, we note that the compressed DCS approach does not address the limitation that scaling DCS for imaging will require many expensive photon-counting APDs. Some recent work [40] has addressed this limitation with parallelized photon-counting measurements with APD arrays for imaging applications. The compressed DCS approach can be readily adopted for such instruments.

## VI. CONCLUSION

We have reported the development of a lossless data compression scheme for fast sampling of photon counts that can be applied to deep tissue blood flow measurements with DCS. We have validated this approach against conventional DCS instruments in experiments on tissue simulating phantoms and in vivo in humans. Our results show that performance of the compressed DCS instrument meets benchmarks set by conventional DCS instruments, while offering lossless data compression of 87.5%.
